# The Anaphase Promoting Complex/Cyclosome Subunit 11 and Its Role in Organ Size and Plant Development

**DOI:** 10.3389/fpls.2021.563760

**Published:** 2021-11-23

**Authors:** Rodrigo Porto Schwedersky, Marina de Lyra Soriano Saleme, Ingrid Andrade Rocha, Patricia da Fonseca Montessoro, Adriana Silva Hemerly, Nubia Barbosa Eloy, Paulo Cavalcanti Gomes Ferreira

**Affiliations:** ^1^Laboratorio de Biologia Molecular de Plantas, Instituto de Bioquímica Médica Universidade Federal do Rio de Janeiro, Rio de Janeiro, Brazil; ^2^Department of Biological Sciences, Escola Superior de Agricultura ‘Luiz de Queiroz’, University of São Paulo, Piracicaba, Brazil

**Keywords:** plant development, cell cycle, anaphase promoting complex (APC), organ size, meristem cells

## Abstract

The anaphase promoting complex/cyclosome (APC/C), a member of the E3 ubiquitin ligase family, plays an important role in recognizing the substrates to be ubiquitylated. Progression of anaphase, and therefore, of the cell cycle, is coordinated through cyclin degradation cycles dependent on proteolysis triggered by APC/C. The APC/C activity depends on the formation of a pocket comprising the catalytic subunits, APC2, APC11, and APC10. Among these, the role of APC11 outside the cell division cycle is poorly understood. Therefore, the goal of this work was to analyze the function of APC11 during plant development by characterizing *apc11* knock-down mutant lines. Accordingly, we observed decreased *apc11* expression in the mutant lines, followed by a reduction in meristem root size based on the cortical cell length, and an overall size diminishment throughout the development. Additionally, crosses of *apc11-1* and *amiR-apc11* with plants carrying a WUSCHEL-RELATED HOMEOBOX5 (WOX5) fluorescent marker showed a weakening of the green fluorescent protein-positive cells in the Quiescent Center. Moreover, plants with *apc11-1* show a decreased leaf area, together with a decrease in the cell area when the shoot development was observed by kinematics analysis. Finally, we observed a decreased APC/C activity in the root and shoot meristems in crosses of pCYCB1;1:D-box-GUS with *apc11-1* plants. Our results indicate that APC11 is important in the early stages of development, mediating meristematic architecture through APC/C activity affecting the overall plant growth.

## Introduction

As sessile organisms, plants achieve growth through postembryonic development to cope with unforeseen environmental conditions. An important strategy is to regulate the balance of cell division and cell differentiation (Horiguchi et al., 2006). For that, cell proliferation and cell expansion must be coordinated, and perfectly followed by cell differentiation progression. This balance is accomplished through the presence of control systems at the division phases: gap one (G1), DNA synthesis (S), gap two (G2), and mitosis (M) to promote mitotic cell cycle or endoreplication, a detour in which the DNA is synthesized, but cell division does not occur (Edgar et al., 2014). However, in the context of multicellular organisms, unlike other eukaryotes, plants control these processes especially in the regions designated as meristems, both on the root apical meristem (RAM) and the shoot apical meristem (SAM) (Steeves and Sussex, 1989; Scheres, 1997).

Meristems are tissues with the capacity for perennial growth. Furthermore, they can develop a plethora of plant organs (Meyerowitz, 1997). However, meristematic cell identity comes from autoregulatory signals that promote a spatial identity and a unique architecture (Van den Berg et al., 1997). The basic meristematic structure in both SAM and RAM is composed of cells in the center known as the mitotically relatively inactive organizing center (OC, SAM) and quiescent center (QC, RAM), surrounded by cells that can divide and progressively differentiate (Galinha et al., 2007; Somssich et al., 2016). The main signals regulating SAM and RAM cell proliferation and differentiation are the CLAVATA3 (CLV3)-WUSCHEL (WUS) signaling pathway. It is summarized by a negative feedback loop in which differentiation-promoting peptide, CLV3 inhibits the expression of the stem cell-promoting transcription factor, WUS cells outside of OC (Somssich et al., 2016). On the other side, PLETHORA (PLT) and SCARECROW (SCR) can interact to promote WUSCHEL-RELATED HOMEOBOX5 (WOX5), the WUS homolog in RAM, expression in QC (Shimotohno et al., 2018). Moreover, WOX5 can establish quiescence in cells by inhibiting CYCLIN D3;3 (CYCD3;3), a key cell cycle transition regulator (Forzani et al., 2014).

The transitions through phases of the cell cycle are regulated by cyclin-dependent kinases (CDKs) activity, with plants having a complex set of CDKs and cyclins when compared to yeast and animals (Komaki and Sugimoto, 2012). CDKA and CDKB are largely responsible for cell cycle control. Interaction studies indicate that CDKA;1 is a key regulator of G1-S transition when associated with D-type cyclins. Downstream on the cycle, CDKA;1 interacting with CYCD3 allows for the progression to M-phase (Dewitte et al., 2007; Boruc et al., 2010; Van Leene et al., 2010; Zhao et al., 2012). On the other hand, CDKB is known to interact mostly with B-type cyclins and CYCA2 to control G2 to M transition and M progression (Boudolf et al., 2009; Vanneste et al., 2011).

Cyclin accumulation is imperative for correct plant cell division (Wolgemuth, 2011). One important system restraining cyclin abundance is the ubiquitin proteasome system (UPS), which controls protein degradation of the major cell cycle regulators. The UPS works through a series of enzymatic reactions carried out by E1 ubiquitin activating, E2 ubiquitin conjugating, and E3 ubiquitin ligase enzymes. The E3 type ligases are key to these processes, selecting the target proteins for ubiquitination (Nowack et al., 2012).

In plants, the following two families of E3 ligases control DNA replication and cell division: SCF (Skp1, Cullins, and F-box proteins) and anaphase promoting complex/cyclosome (APC/C) (Genschik et al., 2014). The APC/C is the largest E3 ubiquitin ligase found in all organisms (Primorac and Musacchio, 2013). Its function depends on essential subunits, such as APC2 and APC11, which together with APC10 and APC3 serve as a docking site for the coactivators, cell division cycle 20 (CDC20), or cell cycle switch52 (CCS52). Coactivators consequently interact with the inhibitors, UV-B-insensitive 4 (UVI4) and omission of second division (OSD). Besides these subunits, APC1, APC4, APC5, APC6, APC7, and APC8 are parts of the multisubunit enzyme backbone (Eloy et al., 2015).

However, the function and activity of APC/C can display multiple dimensions. First, the genes encoding the subunits are differentially expressed through plant development (Eloy et al., 2006). Additionally, coactivator, APC/C^CDC20^ is present in the early G2-M phase and cell cycle exit, while APC/C^CCS52A^ is expressed in late S and G2 phases (Heyman and De Veylder, 2012). Furthermore, coactivator, CCS52A and APC3, encode two genes in *Arabidopsis* with distinct functions and localization during plant development (Blilou et al., 2002; Vanstraelen et al., 2009). Another subunit encoded by multiple isoforms is CDC20, although, only CDC20-1 and CDC20-2 are the predominantly activating APC/C, being responsible for the control of cell number (Kevei et al., 2011). Additionally, lack of expression and the unusual gene structure of the isoforms from CDC20-3 to CDC20-5, suggest that they are pseudogenes (Heyman and De Veylder, 2012).

Finally, another feature on the APC/C function is derived from its targets. Among them are important cell cycle regulators in anaphase promotion, such as Securin and cyclin B (Guo et al., 2016). The recognition of its target mainly occurs *via* D-box or KEN-box protein motifs (Genschik et al., 1998). Securin poly-ubiquitination is required for sister chromatid separation and cyclin B degradation is required for correct cell division afterward (Peters, 2006). Additionally, the time of cyclin B degradation is crucial, since B-type cyclin-dependent kinase CDKB1;1 inhibition induces mitotic progression through the disassembly of the mitotic spindle, chromosome decondensation, cytokinesis, and restoration of the nuclear envelope (Genschik et al., 2014). Furthermore, APC/C^CCS52A^ has been investigated for potential targets through tandem affinity purification (Van Leene et al., 2010). The data identified, among others, the transcription factor, ERF115 as a target that is rate limiting for cell divisions on RAM QC (Heyman et al., 2013).

Although much has been learned about APC/C function in recent years, functional characterization of its essential subunit, APC11 is still lacking. In this study, we have analyzed *apc11* knock-down mutants, which exhibit smaller organs and disturbance of cell cycle gene markers expression. Furthermore, *apc11* plants show an impairment of root architecture in crosses with plants carrying a WOX5 promoter-driven fluorescent marker. Moreover, *apc11* plants show a reduced leaf area together with a decrease in the cell area, when shoot development was observed through the time by kinematics assay. Finally, we observed a diminished APC/C activity in the RAM and SAM through crosses between *pCYCB1;1:D-box*, and *apc11* plants. Our results indicate that APC11 is important during the early stages of plant development by mediating meristematic architecture through APC/C activity, leading to an overall decrease in the plant growth.

## Materials and Methods

### Plant Material and Production of Transgenic Plants

Two T-DNA insertions lines, located in the third exon, and into the 3′ UTR of *APC11* gene were obtained from the Nottingham Arabidopsis Stock Centre (NASC).^1^ The seed code for the lines was as follows: SALK_019654 (*zyg1-2* Guo et al., 2016) and SALK_046847.33.70.x (*apc11-1)*. The presence of the T-DNA insertion was confirmed by a genomic PCR from the leaves of 15-day-old plants. Plants were grown *in vitro* in half-strength Murashige and Skoog (MS) medium (Murashige and Skoog, 1962) supplemented with 1% sucrose, or on soil under long-day conditions (16 h light, 8 h darkness) at 22°C. For all analyses, the *Arabidopsis thaliana* (L.) Heyhn accession Columbia (Col)-0 was used as wild type (WT).

For the *APC11* promoter analysis, a 3.5-kb genomic fragment (upstream of the ATG start codon) containing the putative *APC11* promoter was amplified from the genomic DNA of *Arabidopsis* plants and cloned into the pDONR201 vector (Invitrogen Corporation, CA, United States) and then transferred to pKGWFS7 destination vector (Karimi et al., 2002) *via* MultiSite LR Clonase reaction (Invitrogen Corporation, CA, United States). Thus, the construct was inserted by floral dip transformation (Clough and Bent, 1998) and eight independent homozygous lines were selected.

For the production of amiRNA-*apc1 1 Arabidopsis* transgenic plants, *Agrobacterium tumefaciens* strain GV3101 harboring the plasmid pMP90 was used for plant transformation using the floral dip method (Clough and Bent, 1998). The amiRNA*apc11* (miR) precursor included in the pRS300 vector was modified by directed PCR mutagenesis (Schwab et al., 2006) and introduced into the pK7WG2 vector (Karimi et al., 2002). Four independent lines were selected by seed germination on MS medium supplemented with kanamycin (50 μg ml^–1^), under long-day conditions (16 h light, 8 h darkness) at 22°C and tested for single locus segregation.

### RNA Extraction and Real-Time Quantitative Reverse-Transcription PCR

For the RT-qPCR, three biological replicates of a pool of six plants were collected for each time point analyzed. The *t*-test was used to analyze the significance of the data obtained. Total RNA was extracted from the frozen material according to Walker and Lorsch (2013). To eliminate the residual genomic DNA present in the preparation, the RNA was treated by RNAse-free DNase according to the instructions of the manufacturer (GE Healthcare).^2^ For the RT-qPCR with roots and leaves, about 25 plants were harvested at 8 days after sowing (DAS) for three biological replicates, and RNA extraction was performed.

Complementary DNA was performed with the SuperScript III first-strand synthesis system (Invitrogen Corporation, CA, United States) with the oligo (dT) primers solution according to the instructions of the manufacturer. Primers were designed with Primer Express Software v.2.0 (Applied Biosystems, CA, United States). The primer sequences used in the qPCR experiments are listed in Supplementary Table 1. The complementary DNA (cDNA) was amplified on an Applied Biosystems 7500 Real-Time PCR System in 96-well plates with Power SYBR^TM^ Green PCR Master Mix (Thermo Fischer Scientific, MA, United States) according to the recommendations of the manufacturer. Melting curves were analyzed to check the specificity of the primer. Normalization was done against the average of the housekeeping genes, UBQ10 and GAPDH: DCt = Ct (gene) Ct [mean (housekeeping genes)] and DDCt = DCt (control) DCt. The DCt values for the three biological replicates were used for statistical analysis; Ct refers to the number of cycles at which SYBR Green fluorescence reaches an arbitrary value during the exponential phase of the cDNA amplification. The data were first normalized to the expression level of the housekeeping genes for each RNA sample and then scaled to the WT expression per gene that was fixed to 1.

### β-Glucuronidase Intensity Quantification and Staining

From eight independent lines selected for promoter analysis, two representative independent lines were used in this study. Seeds were plated on MS medium, and after 3 days at 4°C, the plates were placed in a growth chamber (22°C; 16 h photoperiod) for 6, 8, or 10 days to measure β-glucuronidase (GUS) staining. For later analysis, plants were grown on the soil until the organ analyzed was developed. Seedlings of p*APC11*:GUS were harvested and incubated in acetone for 10 min and, subsequently, incubated in 5-bromo-4-chloro-3-indolyl-b-glucuronide (X-Gluc) buffer [100 mM2-amino-2-(hydroxymethyl)-1,3-propanediol (TRIS)–HCl, 50 mM NaCl buffer (pH 7.0), 2 mM K3[Fe(CN)6], and 4 mM X-Gluc] at 37°C for 5 h or 24 h. Seedlings were washed in 100 mM TRIS–HCl, 50 mM NaCl (pH 7.0) and cleared overnight in 95% ethanol, then kept in 90% lactic acid. Samples were photographed under a differential interference contrast (DIC) microscope or stereo light microscope (Leica, Wetzlar, Germany). To quantify the intensity of the GUS staining, the stained area of each RAM and SAM of *apc11*.*1 × CYCB1*,*1*:D-box-GUS and WT × *CYCB1*,*1*:D-box-GUS (Eloy et al., 2011) was marked and its intensity was measured and quantified with the ImageJ software with the values given in arbitrary units. Approximately, 15 plants were analyzed for each experiment.

### Root Growth Analysis

Seeds were plated on a half-strength MS agar growth medium and placed at 4°C for 3 days to synchronize germination. Plates were then placed vertically in a growth chamber (22°C; 16 h photoperiod). Root growth was visualized through photographs taken at 8, 10, and 12 DAS. The root length of at least 22 plants per experiment was measured with the ImageJ software. The root meristem length from the quiescent center (QC) to the first elongated cell exhibiting vacuolization was measured at 7 DAS from around 15 ± 2 roots of *apc11*, *amiR-apc11*, and WT seedlings for each experiment. The samples were visualized with Microscope Axio Imager.A2 (Carl Zeiss AG, Jena, Germany) using a DIC objective. Photographs of roots were used to measure the meristem length with ImageJ software. Lateral root density was scored as the lateral root number per centimeter of primary root and was calculated by dividing the number of lateral roots by the primary root length for each seedling (13 ± 1 seedlings were analyzed).

*For apc11* × p*WOX5*:ERGFP (Xu et al., 2006) crosses analysis, *Arabidopsis* roots of 12 seedlings were imaged between the slide and coverslip on a Confocal Laser Scanning Microscope (Carl Zeiss AG, Jena, Germany). Excitation was done with a multi-argon laser (458, 488, and 514 nm). Fluorescence was detected through a spectral emission window ranging from 493 to 598 nm. The *amiR-apc11* × p*WOX5*:ERGFP crosses analysis was done using a Fluorescence Microscope Axio Imager.A2 (Carl Zeiss AG, Jena, Germany); about 15 roots were visualized under green fluorescent protein (GFP) filter.

Starch granules in the root tips were stained with Lugol’s solution for 5–7 min, then mounted on slides with chloral hydrate, and checked immediately; about 20 roots of 6 DAS for each genotype were analyzed.

### Shoot Growth Analysis

Plants were grown *in vitro* in a half-strength MS medium (Murashige and Skoog, 1962) at 22°C, under 16 h photoperiod. For the measurements of rosette leaf size, six seedlings were grown on the soil until its full life cycle ≈60 DAS; photographs were taken in 25, 30, and 50 DAS. Individual rosettes were measured with ImageJ software.

### Kinematic Analysis

The complete kinematics was analyzed as described (De Veylder et al., 2001) on leaves 1 and 2, from 4 or 5 *apc11*.*1*, and WT plants grown *in vitro* harvested daily from 7 to 22 DAS. The leaves were cleared with 100% ethanol, mounted in lactic acid on microscope slides, and photographed. The leaf area was determined with the ImageJ software.^3^ Abaxial epidermal cells of four to five blades of leaves 1 and 2 were photographed and drawn. Photographs of leaves and drawings were used to measure the leaf area and to calculate the average cell area, respectively, with the ImageJ software. Leaf and cell areas were subsequently used to calculate the cell numbers and cell division rate.

## Results

### *APC11* Expression Through Development and Organs

To investigate the APC11 function during plant growth and development, we analyzed two T-DNA insertion lines. For the *SALK_019654* (*zyg1-2*), we confirmed the heterozygous insertion located in the third exon according to that described by Guo et al. (2016). However, for this study, we obtained an extra line, *SALK_046847*.*33*.*70*.*x*, hereafter referred to as *apc11-1*. In contrast with *zyg1-2*, a homozygous mutant was found harboring a T-DNA insertion located in the 3′ UTR; although *APC11* expression was not completely abolished in this homozygous mutant, instead, the knock-down expression was observed. Additionally, amiRNA*APC11* (amiR*-apc11*) mutants were generated. Four independently transformed plants were obtained, and three representative lines expressing the construct and showing downregulation of *APC11* were analyzed (Supplementary Figure 1).

**FIGURE 1:**
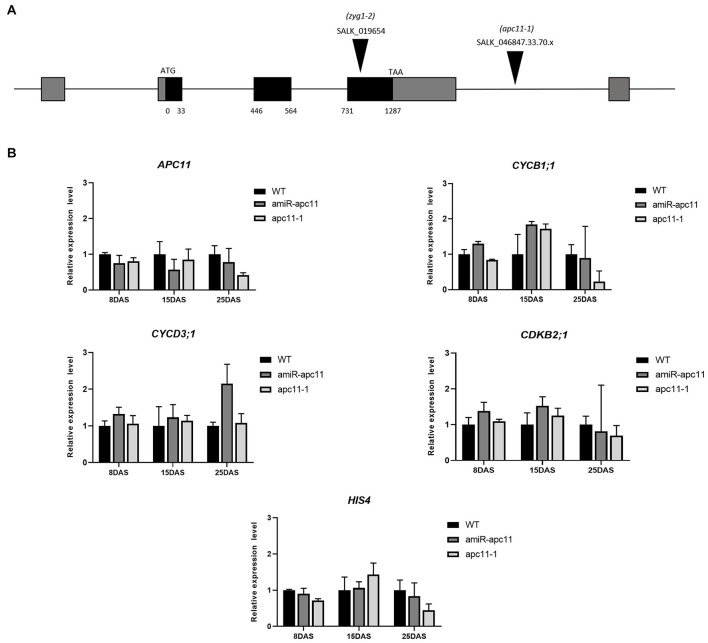
The *apc11* mutant line analysis through development. For functional analyses, SALK lines accessions, 019654 (*zyg1-2*), 046847.33.70.x (*apc11-1*), and an amiRNA *APC11* construct (amiR*-apc11*) were used. **(A)** Map of the genomic region of *APC11* showing the T-DNA insertion site of the SALK mutants. **(B)** Quantitative RT-PCR transcript analysis of *APC11* and cell cycle gene expression markers in *apc11-1*, *amiR-apc11* mutants, and wild-type (WT) plants. Total RNA was prepared from the whole seedlings harvested at 8, 15, and 25 days after sowing (DAS) and amplified by qRT-PCR. All values were normalized against the expression level of the housekeeping genes and expression compared to the expression data in the WT control. Data are means ± SD (*n* = 3).

To assess the *APC11* mRNA levels in the mutants selected, the expression profile of whole seedlings was analyzed by RT-qPCR on 8, 15, and 25 DAS. In agreement with the published data (Guo et al., 2016), the *APC11* expression was not completely abolished in the T-DNA mutants analyzed (*apc11-1* and *zig1-2*), confirming the essential nature of APC11 for plant development. Instead, we observed a reduced *APC11* mRNA expression level on both T-DNA mutant lines (Supplementary Figure 1B), as well as in the *amiRNA-apc11* lines compared to the wild type (WT), Columbia-0 in all time points analyzed (Figure 1B and Supplementary Figure 1). However, *amiRNA-apc11* showed a pronounced reduction in the expression levels at 15 DAS, while *apc11-1* showed the most prominent reduction at 25 DAS. To carry out the deeper analyses of the effect of *APC11* downregulation in plant growth, we followed our characterization using both homozygous lines, *apc11-1* T-DNA and *amiR-apc11*.

To investigate which phase of the cell cycle is affected by APC11 downregulation, we analyzed the expression levels of selected DNA synthesis (S) and mitosis (M) phase marker genes: *CYCB1;1; CDKB2;1;CYCD3;1*, and HIS4. CYCB1;1 and CDKB2;1 are involved in the control of gap two (G2)-M transition (Hemerly et al., 1992; Segers et al., 1996), while CYCD3;1 and HIS4 are G1/S phase-specific genes (Menges and Murray, 2002; Menges et al., 2003), which also influence the M phase (Schnittger et al., 2002). Our results showed a very similar expression profile of the two G2-M specific genes in both the lines during the time points analyzed, showing slight upregulation on 8 DAS, increasing the expression at 15 DAS, and downregulating their expression levels at 25 DAS (Figure 1B). While the G1/S phase-specific genes, *CYCD3;1* and *HIS4* did not show any significant variation on their transcripts levels in almost all time points analyzed, with the exception for *CYCD3;1* transcript in *amiR-apc11* line at 25 DAS (Figure 1B).

**FIGURE 2:**
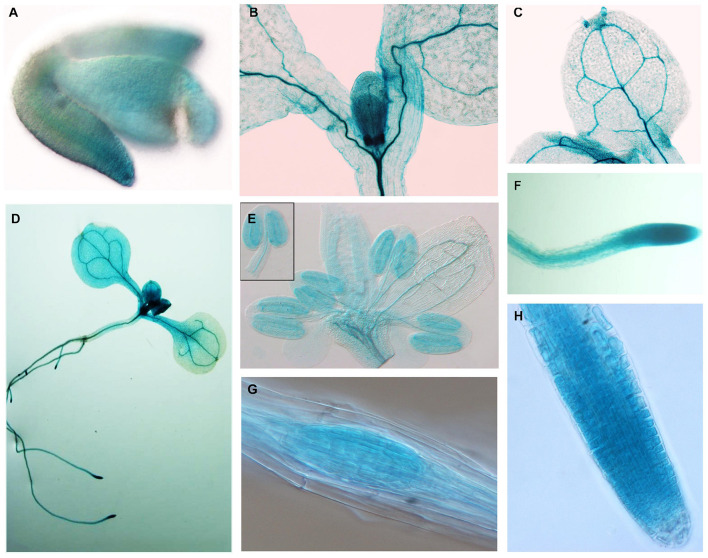
*APC11* tissue expression pattern. Expression of the p*APC11*–GUS reporter gene at different developmental stages of two representative *APC11* promoter lines. **(A)** Embryo showing early expression in the meristematic regions. **(B)** Shoot detail showing staining in the meristematic region. **(C)** Mature leaf showing a positive marker on vasculature system. **(D)** Seedling at 10 DAS. **(E)** Inflorescence at 30 DAS, detail showing the anther. **(F)** The Main root showing staining on the vasculature and meristematic region. **(G)** Secondary root meristem. **(H)** Main root tip at 6 DAS. Meristematic detail shows decreased expression on the elongated cells.

To study the expression pattern of *APC11* in plant tissues, a 3.5-kb fragment upstream to the ATG start codon of *APC11* was fused to a β-glucuronidase (GUS)-GFP tandem reporter cassette and introduced into *Arabidopsis* plants. Staining for β-glucuronidase (GUS) activity revealed a strong and more or less constitutive expression in the whole plant. We observed expression during the initial phase of plant development: at mature embryo, appearing stronger in mitotically active regions of RAM, SAM, cotyledons, and young leaves (Figures 2A,B,D,F, and Supplementary Figure 11A).

Further in development, *APC11* expression is still high in vascular cambium tissues on both the leaves and the roots (Figure 2C and Supplementary Figure 11E). Furthermore, we continue to observe the presence of *APC11* in the meristematic regions of the secondary roots (Figures 2D,G). We also investigated the expression pattern in the floral organs, and we detected expression in floral buds, carpels, stamen, and gynoecium (Figure 2E and Supplementary Figures 11B,C), confirming its importance for the female and male gametogenesis as reported previously (Guo et al., 2016). Together, this data points toward *APC11* function on plant development, mainly in meristematic tissue on both the shoots and the roots. The next step was to further analyze those sites on *apc11* mutants.

To test the possibility that *APC11* is involved in seed development, both T-DNA mutant lines were self-crossed and seed production was analyzed. We observed a reduced viable seed set in *apc11-1* and *zig1-2* lines (Supplementary Figure 2). In *apc11-1* siliques, approximately 30% of ovules did not develop into mature seeds, being aborted compared to WT, which supports the importance of APC11 for seed development. To test male viability on *apc11-1* and *zyg1-2* plants, we utilized Alexander stain (Alexander, 1969) to observe pollen integrity. Interestingly, both *apc11 mutants* do not show any problem in pollen production as seen in anthers colored by intense red (Supplementary Figure 3).

### Phenotypic Analyses of the Roots of *apc11* Mutants

**FIGURE 3:**
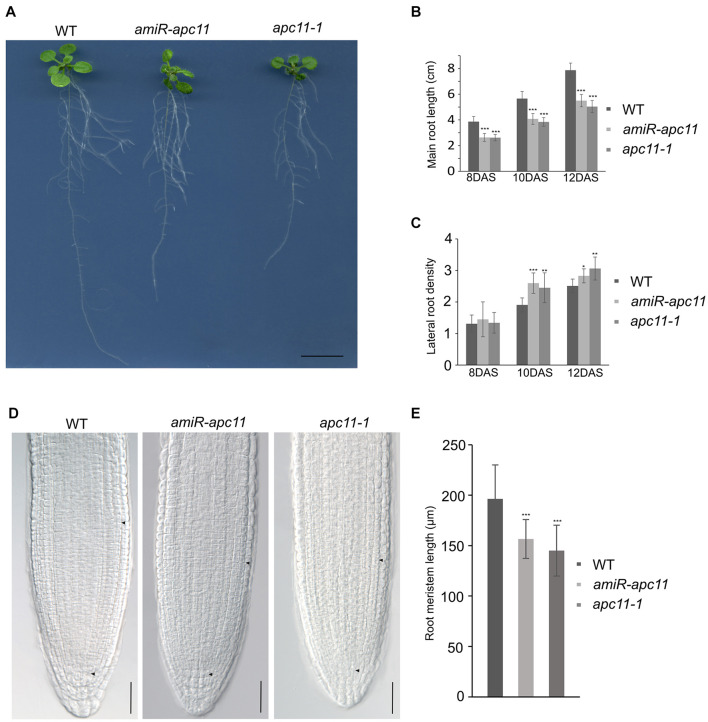
The *apc11-1* and *amiR-apc11* plants develop smaller root sizes. Root development impairment in *apc11* mutant plants observed through time. **(A)** Representative picture of root length in 12 DAS. Black bar = 1 cm. **(B)** Measurement of main root length in *apc11-1*, *amiR-apc11*, and WT plants at 8, 10, and 12 DAS; *n* = 22 (***P value < 0.001, Student’s t test). **(C)** Measurement of the lateral root density at 8, 10, and 12 DAS of *apc11-1*, *amiR-apc11*, and WT plants. *n* = 13 (*P value < 0.05; **P value < 0.01; ***P value < 0.001, Student’s t test). **(D)** Representative meristematic region of the main root tip, DIC image at 7 DAS showing a smaller meristem length. Black bar = 30 mM. **(E)** Measurement of root meristem length at 7 DAS of *apc11-1*, *amiR-apc11*, and WT plants. *n* = 15 ± 2 (***P value < 0.001, Student’s t test).

To investigate the effects of the *apc11* downregulation on root development, we first investigated *apc11-1* and *amiR-apc11* root size throughout the time. The main root length of *apc11-1* and *amiR-apc11* were analyzed at 8, 10, and 12 DAS. As showed in Figure 3, the main root length was statistically shorter in both mutants, during all time points analyzed. Observing the clear expression of *APC11* in the lateral root primordia, we investigated the effect on the lateral root development, by assessing the lateral root density on *apc11* mutants. Based on the main root size and the number of lateral roots, we were able to calculate the lateral root density, and we observed an increase in the root density measurements, especially at 10 and 12 DAS, this difference being significantly higher in both mutants. Additionally, roots from two other amiR-*apc11* lines were evaluated at 12 and 15 DAS displaying also the same phenotype (Supplementary Figure 6). Similarly, measurements of the root length of *zyg1-2* at 10, 12, and 15 DAS (Supplementary Figure 7), showed the same root length phenotype observed with the other *apc11* mutant lines.

The RAM was measured from the QC toward the point where cortical cells start to elongate (black arrow, Figure 3D), and the results were statistically analyzed revealing that *apc11-1* and *amiR-apc11* plants exhibited smaller root meristem size compared to WT as seen in the graphic representation (Figure 3E). These data suggest an important role of APC11 on the root development starting probably before the time point analyzed (8 DAS).

**FIGURE 4:**
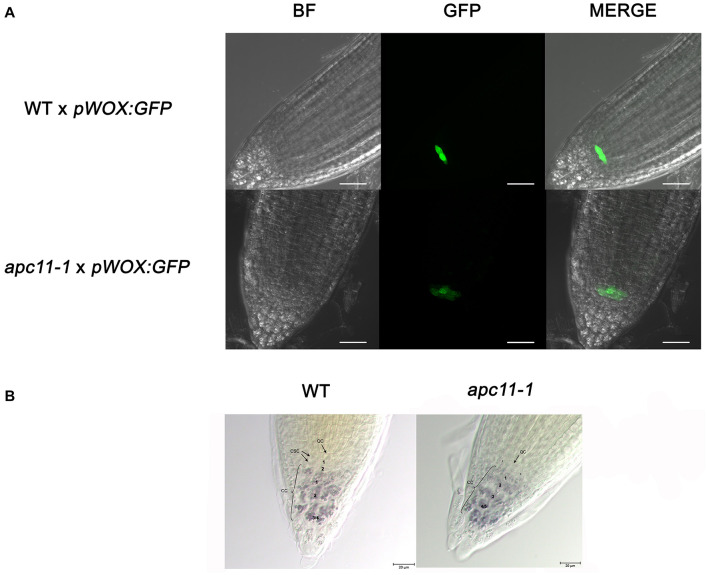
The *apc11*.*1* crosses with *pWOX5*:*erGFP* markers show altered quiescent center (QC). Confocal microscopy of the meristematic architecture of 6 DAS roots of mutant (*apc11-1*) and WT plants crossed with *pWOX5:erGFP*. The middle panel corresponds to green fluorescent protein (GFP) emission. The left panel corresponds to Bright Field (BF) images, and the right panel corresponds to the merged picture of GFP and BF images. White bar = 30 μm. **(A)** WT plants show normal phenotype exhibiting a plane with 4 quiescent cells (QCs), while *apc11-1* mutant shows extended expression of the *pWOX5:erGFP* marker. **(B)** Representative DIC images of the QC cells in the WT and *apc11-1* plants. Lugol’s staining showing the accumulation pattern of starch granules in the columella stem cells (CSCs).* Starch granules indicating the differentiation of CSC. Black bar = 20 μm, *n* = 15.

To pinpoint what is possibly causing the root phenotype, we used the *apc11-1* mutant to cross with the markers lines, *pSCR:YFP* (Koizumi et al., 2012) and *pWOX5:GFP* (Xu et al., 2006). The *SCR* gene is related to formative cell divisions, which originate from the cortex and endodermis, organizing the ground tissue in the roots (Fukaki et al., 1998). The F1 crosses, *apc11-1* × *pSCR:YFP*, did not show visible differences compared to the control crosses, WT × *pSCR:YFP* (Supplementary Figure 8). In contrast, the *WUSCHEL-RELATED HOMEOBOX5* (*WOX5*) gene is expressed specially in the QC, and *apc11-1* crossed with *pWOX5*:*erGFP* plants showed a visible lower expression in 40% of the F1 seedlings (Figure 4). Additionally, we noticed a larger area expressing the WOX5 marker in the mutant, indicating there are more or bigger cells with QC identity. Indeed, when we carefully looked at the QC cells (Figure 4B), we observed a bigger cell area of the QC compared to the control. Additionally, we observed a difference in the pattern of columella stem cells (CSC). As can be seen in Figure 4B, the *apc11*.*1* mutant shows starch granules in the cell layer below the QC, indicating that this layer is a differentiated columella cell (DCC), and not the CSC. To support the data obtained with *apc11-1 × pWOX 5:erGFP*, the *amiR-apc11* line was also crossed with the *pWOX5:erGFP* marker, and the results presented similar outcomes. The crossing displayed lower expression in QC cells compared to the WT (Supplementary Figure 9). The *amiR-apc11* also shows starch granules in the layer below the QC, and therefore these cells are differentiating. However, the cells next to it appear to be devoid of starch, suggesting that some properties of the stem cells remain. This would be in agreement with the QC-restricted expression of *WOX5:erGFP*.

Together, our study on the role of APC11 in root development points toward an early effect of APC/C during the root development leading to a shorter RAM and overall root size.

To exclude the possibility that the observed phenotype on *apc11* roots is due to a disturbance in auxin distribution or localization, we tested the effect of *APC11* downregulation in the DR5 auxin responsive promoter (Sabatini et al., 1999). Our *apc11-1* mutant crossed with *pDR5:RFP* plants showed no effect in auxin response (Supplementary Figure 10).

### Role of *APC11* in Shoot Development

To address the effects of *apc11* mutation in shoot development, rosettes from *apc11-1* and *zyg1-2* were measured through development (Supplementary Figure 4). The data obtained showed a similar decrease in rosette size of the two *apc11* T-DNA mutant plants at 15, 35, and 50 DAS (Supplementary Figure 5A). The difference can be seen through consecutive time points (Supplementary Figures 4B, 5A), suggesting that the source of the observed phenotype may be from previous stages of development. Such observation implies that *apc11-1* and *zyg1-2* plants did not manage to circumvent the APC/C impairment in the early development stages, leading to smaller plants in the final stages of development.

To shed light on the question about the difference observed in the rosette area during later development stages, and the cellular basis of this trait, the daily quantitative image of the first leaf pair were obtained from 7 to 22 DAS and used to measure the leaf blade area, the cell area, and the cell number of the abaxial epidermis from the *apc11-1* mutant. Together, the data were used to evaluate the cell division rate (CDR) (De Veylder et al., 2001).

**FIGURE 5:**
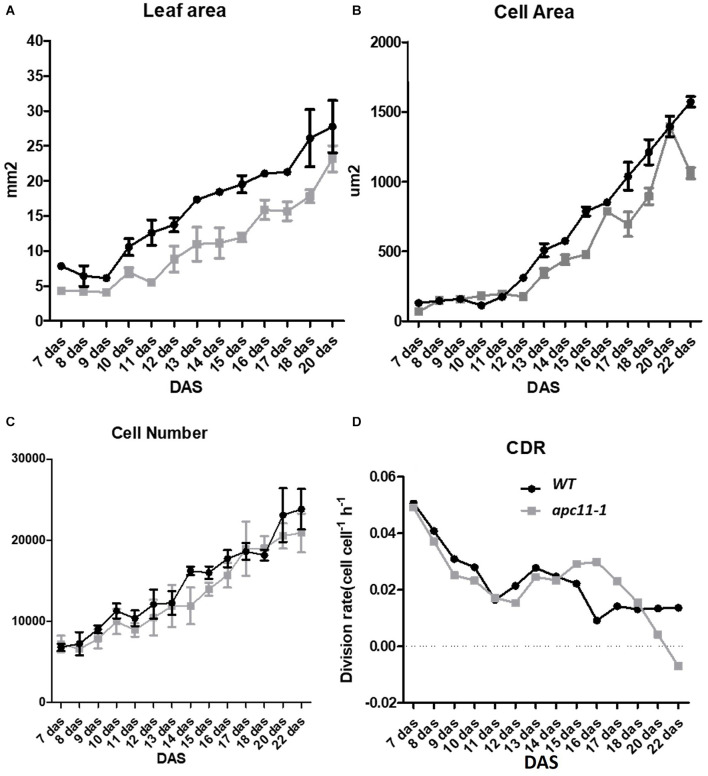
Kinematics analysis of *apc11-1* (gray square) and WT (black circle) plants grown *in vitro*. **(A)** Leaf blade area. **(B)** Cell area. **(C)** Cell number quantification on the abaxial side of the leaves. **(D)** Cell division rate (CDR).

In agreement with the reduced rosette phenotype observed, in the first days of leaf development, the *apc11-1* plants already displayed a smaller leaf area. This difference increased through 10 DAS, and at 13 DAS, leaves in the mutants were about 40% smaller than in the WT plants. At the mature stage, the *apc11-1* plants exhibited leaf blade area only 25% smaller (Figure 5A). Cellular measurements showed a similar cell number overall in the two groups, ranging from 7 × 10^4^ up to 2 × 10^5^ (Figure 5C). The average cell size in the control plants and *apc11-1* mutant was approximately the same from 7 to 11 DAS (Figure 5B). However, after 12 DAS onward, we observed an average smaller cell area on *apc11-1* mutant compared to the WT, which became more pronounced at 15 DAS. Additionally, CDRs were calculated based on the cell number measurements, showing higher values between days 15 and 17 in the *apc11-1* (Figure 5D), and significantly dropping after this period, which is in agreement with what was observed in the expression profile of *CYCB1;1* and *CDKB2;1*, increase at 8 and 15 DAS, followed by a decrease at 25 DAS (Figure 1B). Taken together, the kinematics analyses indicate the influence of APC11 on leaf growth. Such difference is mainly due to a decrease in the cell area, with no difference in cell numbers, leading to a significant decrease in the CDR after 17 DAS.

### The Anaphase Promoting Complex/Cyclosome-Mediated CYCB1;1 Degradation on *apc11* Plants

**FIGURE 6:**
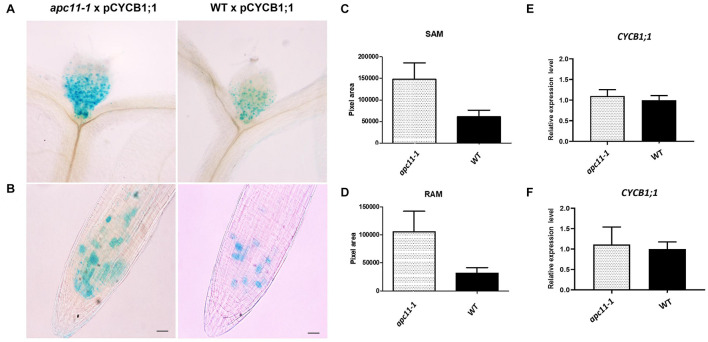
Meristematic loss of function of APC in *apc11-1* viewed through p*CYCB1;1:*D–box– β-glucuronidase (GUS) crosses. Anaphase–promoting complex (APC) activity in mutant (*apc11 −/−*) plants crossed with p*CYCB1;1*:D–box–GUS construct. **(A)** Picture of the p*CYCB1;1*:D–box–GUS staining on leaves 1 and 2 at 8 DAS of mutant and WT lines. **(B)** Picture of the p*CYCB1;1*:D–box–GUS staining on roots at 8 DAS of mutant and WT lines. **(C)** Measurement graph of GUS intensity on leaves from the mutant and WT lines; *n* = 15, *P* < 0.0001. **(D)** Measurement graph of GUS intensity on roots from the mutant and WT lines; *n* = 15, *P* < 0.001. **(E)** Quantitative RT-PCR transcript analysis of *CYCB1;1* gene expression marker in *apc11-1* and WT from leaves at 8 DAS. All values were normalized against the expression level of the housekeeping genes and expression compared to the expression data in the WT control. Data are means ± SD (*n* = 3). **(F)** Quantitative RT-PCR transcript analysis of *CYCB1;1* gene expression marker in *apc11-1* and WT from roots at 8 DAS. All values were normalized against the expression level of the housekeeping genes and expression compared to the expression data in the WT control. Data are means SD (*n* = 3).

Although our data pinpointed APC*11* expression on meristems through GUS staining and involvement in both RAM and SAM development, a question regarding APC/C activity on these tissues still remained. To address it, we crossed p*CYCB1;1*:D-box–GUS with *apc11-1* plants. The p*CYCB1;1*:D-box–GUS construct is expressed only in G_2_/M transition and contains D-box, ensuring APC/C targeting proteolysis, and enabling quantitative analyses of transient mitotic and indirect APC/C activity (Colón-Carmona et al., 1999). We used p*CYCB1;1*:D-box–GUS crossed with WT as a control. Both SAM and RAM of 15 F_1_ plants from each cross (*apc11*.*1* × p*CYCB1;1*:D-box–GUS and WT × p*CYCB1;1*:D-box–GUS) were collected, stained for GUS activity, and cleared with lactic acid. Magnified photographs were taken at 8 DAS and images were analyzed on ImageJ software (see “Materials and Methods”). The GUS activity was positive in both groups in the meristem tissues (Figure 6). However, at 8 DAS, SAM and RAM patterns of *apc11* × p*CYCB1;1*:D-box–GUS crosses show more cells expressing the marker (*p* < 0.0001) with GUS staining positive cells. To exclude the possibility that the higher protein accumulation observed is due to the increased *CYCB1;1* expression, rather than the increased protein stabilization, the expression levels of *CYCB1;1* was checked by RT-qPCR in roots and leaves of *apc11-1* and WT plants at 8 DAS (Figures 6E,F). As shown in Figures 6E,F, the mRNA levels of *CYCB1;1* were not changed at this specific time point, which strongly suggests that the knock-down of the *APC11* gene causes a decrease in the D-box-dependent proteolysis of CYCB1;1.

## Discussion

The anaphase promoting complex/cyclosome is a large multisubunit complex highly conserved through species (Capron et al., 2003; Eloy et al., 2011; Wang et al., 2012). As an enzyme that ubiquitylates target proteins for cell cycle progression, its main function is to recognize and mark proteins fated to degradation by UPS. The ability to ubiquitylate substrates depends on essential subunits, APC2 and APC11 (Tang et al., 2001), which together with APC10 and APC3 serve as a docking site for coactivators cell division cycle 20 (CDC20) or cell cycle switch52 (CCS52), that comprise the catalytic core (Heyman and De Veylder, 2012).

In this study, we provide insights into the functional characterization of the essential APC/C subunit, APC11, from *Arabidopsis thaliana*. We approached the function of the gene by using the T-DNA lines available, and generating *APC11* amiRNA lines, all of them showing decreased mRNA levels, confirmed through RT-qPCR. The *APC11* expression was diminished at all time points analyzed (8, 15, and 25 DAS) compared to WT (Figure 1 and Supplementary Figure 1). We also observed the essential nature of APC11; as for the T-DNA lines, we could not restore any homozygous plants from full knock-out, confirming previous results (Guo et al., 2016). Indeed, inactivation of several APC/C subunits is lethal in all organisms studied; mutations in most APC/C subunits affect female and/or male gametogenesis, showing the essentiality of the core subunits (Yu et al., 1998; Yamashita et al., 1999; Cullen et al., 2000; Bentley et al., 2002; Garbe et al., 2004; Pál et al., 2007; Jin et al., 2010; Saleme et al., 2021).

Based on the analysis of the GUS reporter lines, *APC11* was expressed in almost all tissues analyzed. Initially, the GUS staining showed *APC11* promoter activity in cotyledonary stage embryos (Figure 2). At 6 DAS, *pAPC11:GUS* expression was observed in root meristems and secondary roots meristems. Additionally, *pAPC11:GUS* staining was present in both SAM and vascular cambium tissues of leaves. The expression pattern of *APC11* seems to be similar to what was observed for *APC4*, *NOMEGA*, *APC10*, and APC/C coactivator *CDC20* and the APC/C inhibitor, *UVI4* (Kwee and Sundaresan, 2003; Wang et al., 2012; Heyman et al., 2017) with the exception of APC/C coactivator, CDC20 and APC10, which were not expressed in the vascular tissue and at RAM, respectively (Eloy et al., 2011; Kevei et al., 2011). Based on the GUS-staining pattern, *APC11* is present in combination with various APC/C subunits and regulators, indicating that the origin of the observed phenotype depends on the activity of the complex as a whole.

The *apc11* mutants produced short roots as a result of the reduced root meristem, besides higher lateral root density (Figures 3C,E), which the latter being explained by higher expression levels of the cell division marker, *CYCB1;1* till 15 DAS (Figure 1B; Beeckman et al., 2001; Branco and Masle, 2019). Furthermore, *pWOX5:erGFP* plants crossed with *apc11-1* mutant showed weak and diffuse patterns on the QC GFP-WOX5 positive cells in 40% of the progenies (Figure 4 and Supplementary Figure 9) showing a larger area expressing the marker (Figure 4). As shown in Figure 4B, there is a disturbance in the QC cells, which display a bigger cell area compared to the WT plants; moreover, Lugol’s staining indicates that *apc11-1* leads to a differentiation of the CSCs. Interestingly, there is also an extension of WOX5 marker expression in these cells, suggesting that these cells may have an intermediate differentiation status. Furthermore, in the *amiR-apc11* line, starch granules were observed in at least one of the CSCs, suggesting that differentiation of CSCs occurs only sporadically. In this mutant, there was no extent of WOX5 expression, suggesting that the *amiR-apc11* lines do not reduce the *APC11* levels to the same extent observed in the *apc11-1* mutant.

Downregulation of *APC11* can be correlated as an indicator of APC/C impairment observed by p*CYCB1;1*: D-box–GUS × *apc11* crosses. Our data present a clear increase in the number of GUS positive cells on RAM at 8 DAS (Figure 6B), suggesting a defective CYCB1;1 degradation and its later accumulation. Moreover, the accumulation of CYCB1;1 GUS positive cells observed in the p*CYCB1;1*:D-box–GUS × *apc11* SAM crosses (Figure 6A), pinpoints toward an APC/C failure in CYCB1;1 degradation in the SAM as well.

Based on the kinematics analysis, the leaf size of *apc11-1* exhibited differences already at 7–10 DAS (Figure 5A), indicating a common control in the shoot and root size development through APC11. On the other hand, leaf analyses between 7 and 10 DAS do show neither cell number nor cell area alterations (Figures 5B,C). On consecutive days until 13 DAS, the *apc11-1* leaves had 40% less area than WT. Furthermore, after the 13th DAS, the cell area in the *apc11-1* mutant was also compromised in our kinematic analyses (Figure 5B). From 17 to 22 DAS, the *apc11-1* mutants could restore some of the differences in the leaf area, exhibiting only a 25% smaller leaf blade area (Figure 5A). In this regard, cell division compensation could be occurring in *apc11-1* after 15 DAS (Figure 5D). Indeed, at the same time frame, an increasing trend in *CYCB1;1* and *CDKB2;1* expression could be driving such process (Figure 1). It is noteworthy a difference in the division rate observed from 15 to 17 days, showing a higher CDR in the mutant plants; indeed when we observed the expression profile on those plants, both cell cycle markers for division are upregulated at 15 DAS. However, after 17 DAS, the CDR drastically fell down compared to WT, which is again followed by a decrease in the expression profile of *CYCB1;1* and *CDKB2;1*. Although our expression data showed *CYCB1;1* upregulation at 15 DAS, followed by downregulation at 25 DAS. We could speculate that this difference would be more visible if the sampling of the material used for the expression analysis were from the roots or the young leaves.

Our results indicate that it is possible, to a large extent, that defects in APC11 activity interfere with cell expansion, since the cell area in *apc11-1* mutants display a smaller area at several time points (Figure 5B), and less differences in cell numbers compared to WT plants (Figure 5C). A shift of APC/C action through mechanisms influenced by other subunits, such as APC2 and APC7 could explain the compensatory leaf growth effect on later time points (Eloy et al., 2006). Furthermore, as the plant develops, it is expected that cell cycle genes progressively diminish their expression (Eloy et al., 2006) as seen in the transcription profile of *APC11*, consequently lessening the role of APC11 in the phenotypic appearance. In fact, reports on tomato CCS52A downregulation lead to CYCA3;1 accumulation (Mathieu-Rivet et al., 2010) and S-specific controller likely involved in endo-replication, thus resulting in cell expansion (Takahashi et al., 2010). In tomato fruits, in the overexpressing CCS52A, the endoreplication was initially delayed, altering the fruit size, but later in time, a compensatory effect recovers the fruit size (Mathieu-Rivet et al., 2010).

*Apc11* mutant plants showed diminished mRNA levels causing a reduced function of the complex in both SAM and RAM, as a consequence of an impaired APC/C ubiquitination activity leading to deficient proteolysis of CYCB1;1, resulting in a clear protein accumulation (Figure 6). Such impairment of the APC/C brings early effects on embryogenesis, with decreased expression levels of the WOX5 marker in the root QC with more cells expressing it, in addition to a smaller meristem size and overall root length. Further, on plant development, the shoot analyses also show the effect of APC11 knockdown on rosette size, probably interfering with the sophisticated mode of activity of APC/C through its subunit interaction, culminating in reduced cell area, hence smaller leaf size.

In conclusion, our study shows the essential role of APC11 for plant viability, and for the proper maintenance of cell division controlled by the regulation of APC/C activity through the APC11 subunit, which seems to play an important role during leaf and root growth and development.

## Author’s Note

This study is dedicated to the memory of the last author, Paulo Cavalcanti Gomes Ferreira, who passed away on July 22, 2020. He will be greatly missed by many, both inside and outside of the scientific community.

## Data Availability Statement

The original contributions presented in the study are included in the article/Supplementary Material, further inquiries can be directed to the corresponding author.

## Author Contributions

PCGF, NBE, and ASH conceived and designed the experiments. RPS, MLSS, IAR, and PFM performed the experiments. RPS, MLSS, and NBE analyzed the data. RPS, NBE, and PCGF wrote the manuscript. All authors have read and approved the manuscript.

## Conflict of Interest

The authors declare that the research was conducted in the absence of any commercial or financial relationships that could be construed as a potential conflict of interest.

## Publisher’s Note

All claims expressed in this article are solely those of the authors and do not necessarily represent those of their affiliated organizations, or those of the publisher, the editors and the reviewers. Any product that may be evaluated in this article, or claim that may be made by its manufacturer, is not guaranteed or endorsed by the publisher.

## Footnotes

1
http://arabidopsis.info/

2
http://www.gehealthcare.com

3
http://rsb.info.nih.gov/ij
