# Supplementary material for: The Anaphase Promoting Complex/Cyclosome Subunit 11 and Its Role in Organ Size and Plant Development
Source: Front Plant Sci. 2021 Nov 23;12:563760. doi: 10.3389/fpls.2021.563760 (PMC8650582; doi:10.3389/fpls.2021.563760)
Supplement: Supplementary file 1 [file Data_Sheet_1.pdf]

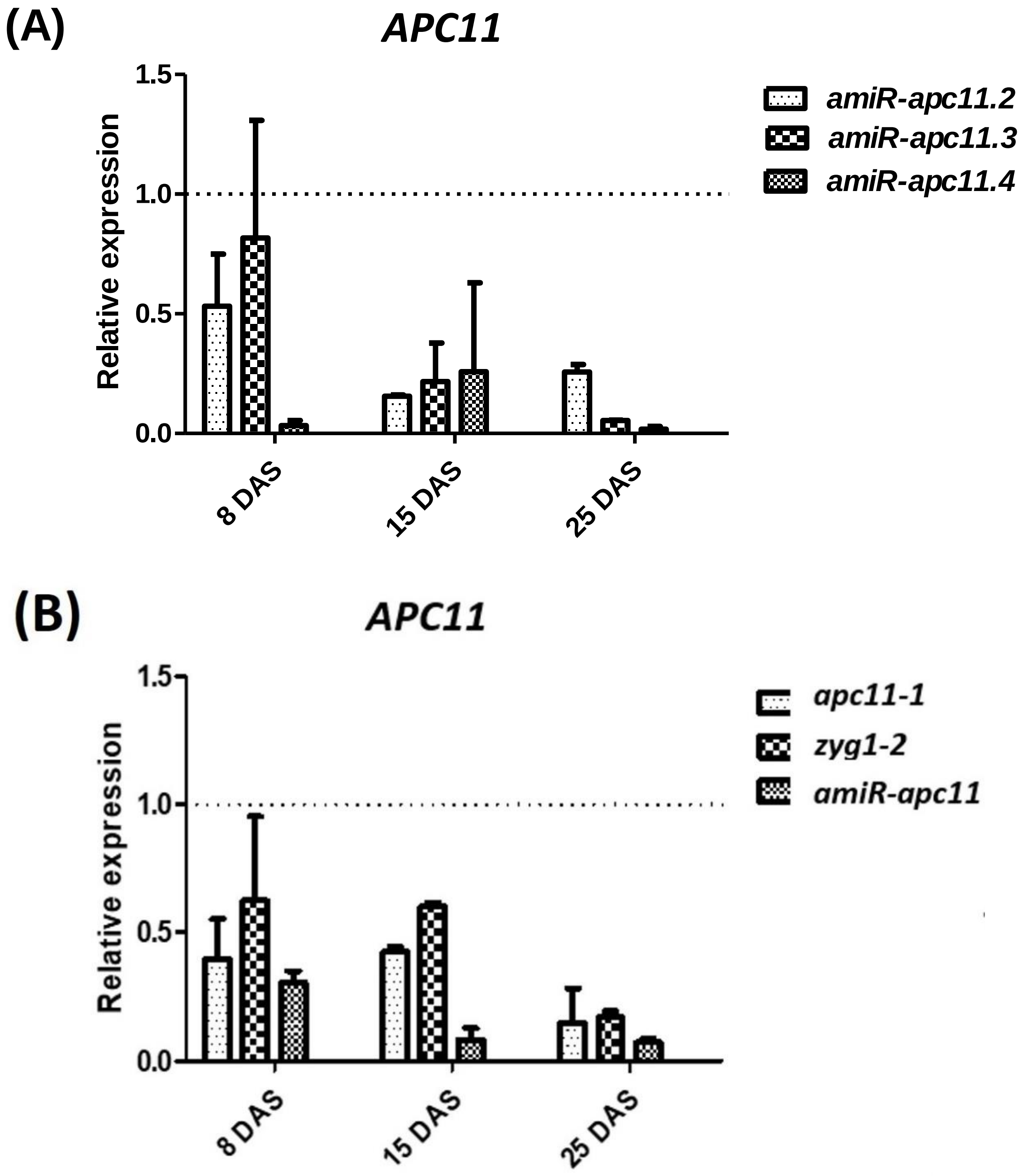

FIGURE S1. Quantitative RT-PCR analysis in differ apc11 mutant plants overall decrease expression through development

(A) RT-qPCR transcript analysis of *APC11* in three independent *amiR-apc11* lines and WT plants. Total RNA prepared from the whole seedlings harvested at 8, 15 and 25 days after stratification (DAS) and amplified by RT-PCR. All values were normalized against the expression level of the housekeeping genes and expression compared to the expression data in the WT control (dotted line). Data are means ± standard deviation (n=3).

(B) RT-qPCR transcript analysis of *APC11* in *apc11-1, zyg1-2 and amiR-apc11* lines and WT plants. Total RNA prepared from the whole seedlings harvested at 8, 15 and 25 days after stratification (DAS) and amplified by RT-PCR. All values were normalized against the expression level of the housekeeping genes and expression compared to the expression data in the WT control (dotted line). Data are means ± standard deviation (n=3).

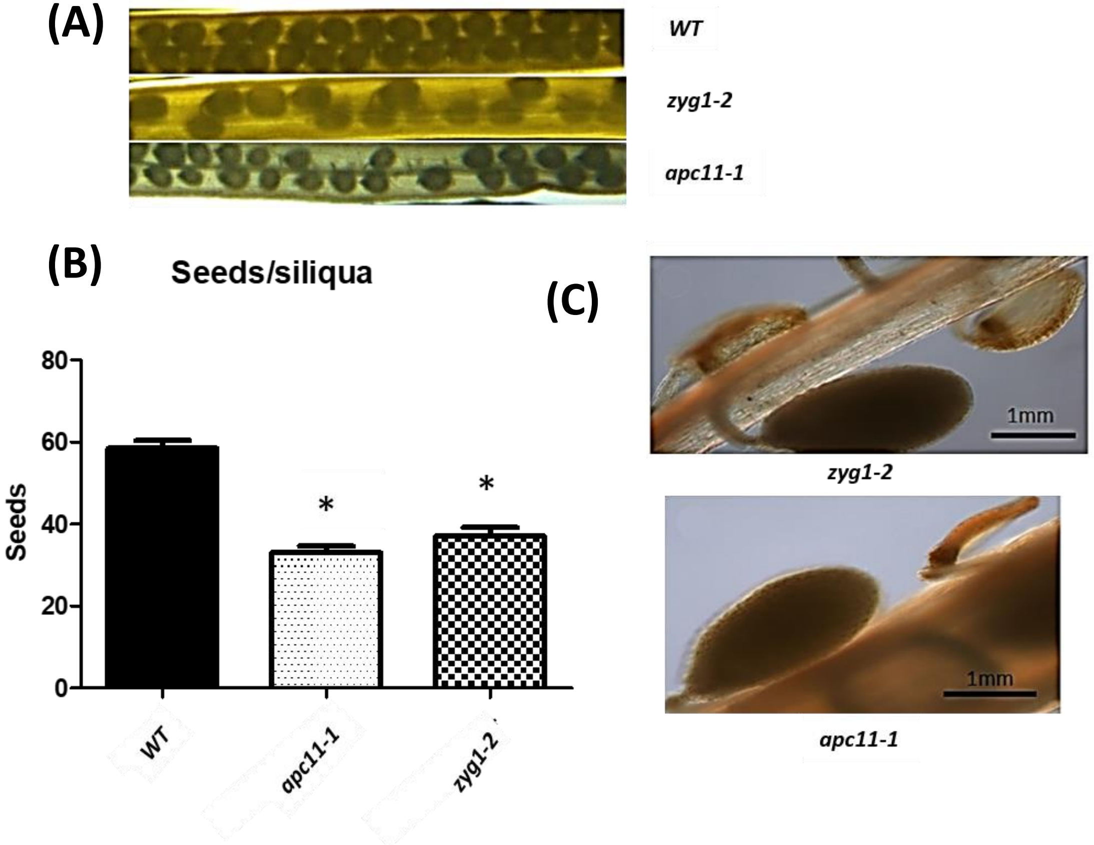

FIGURE S2. *apc11* seed development

Seed development in wild-type and SALK lineages accessions: *zyg1-2 and apc11-1*.

(A) Representative picture of siliques of WT, *zyg1-2* and *apc11-1*.

(B) Graph of seed development per silique of mutant plants, analyzed statistically. N=8. P<0.001(*).

(C) Aborted seeds detail picture of *zyg1-2* (upper) and *apc11-1* (lower).

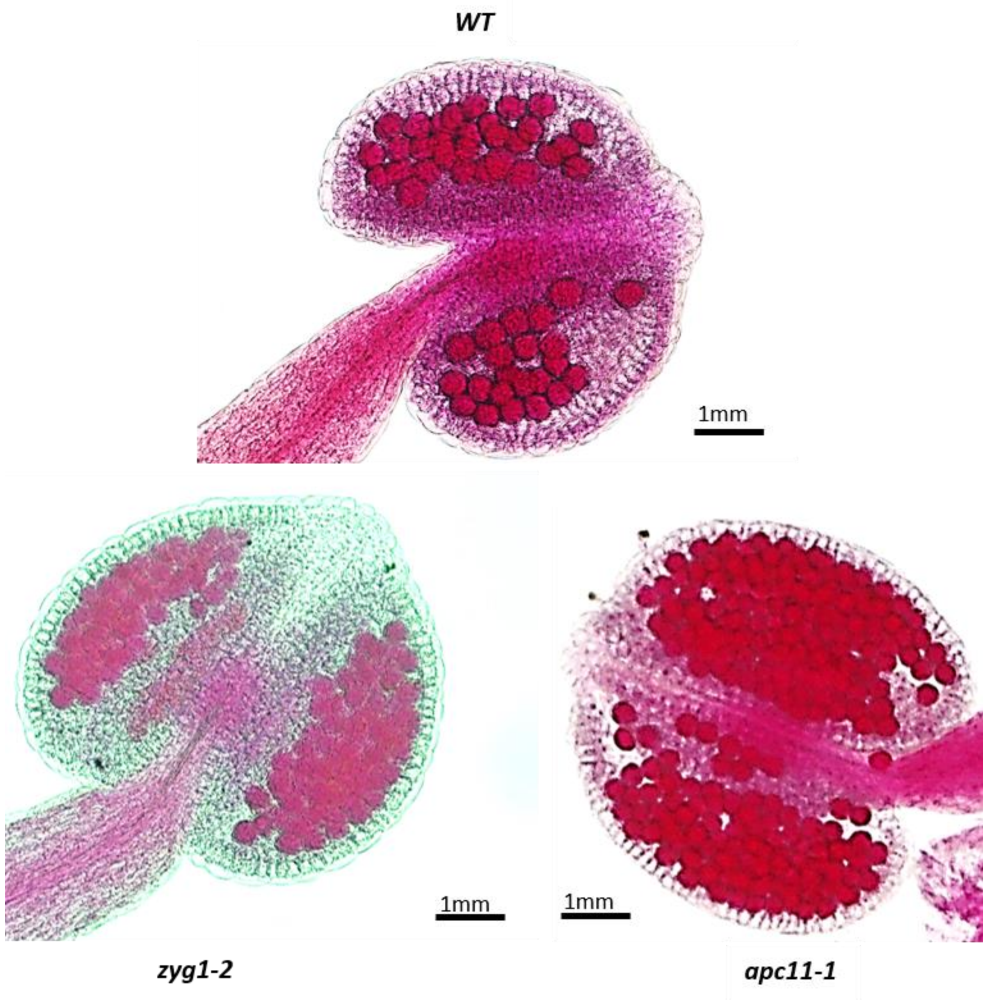

FIGURE S3. *apc11* pollen viability

Pollen integrity in wild-type (upper), *zyg1-2* (lower left), *apc11-1* (lower right). To test for pollen viability on *apc11 mutant* plants we utilized Alexander stain (Alexander, 1969)

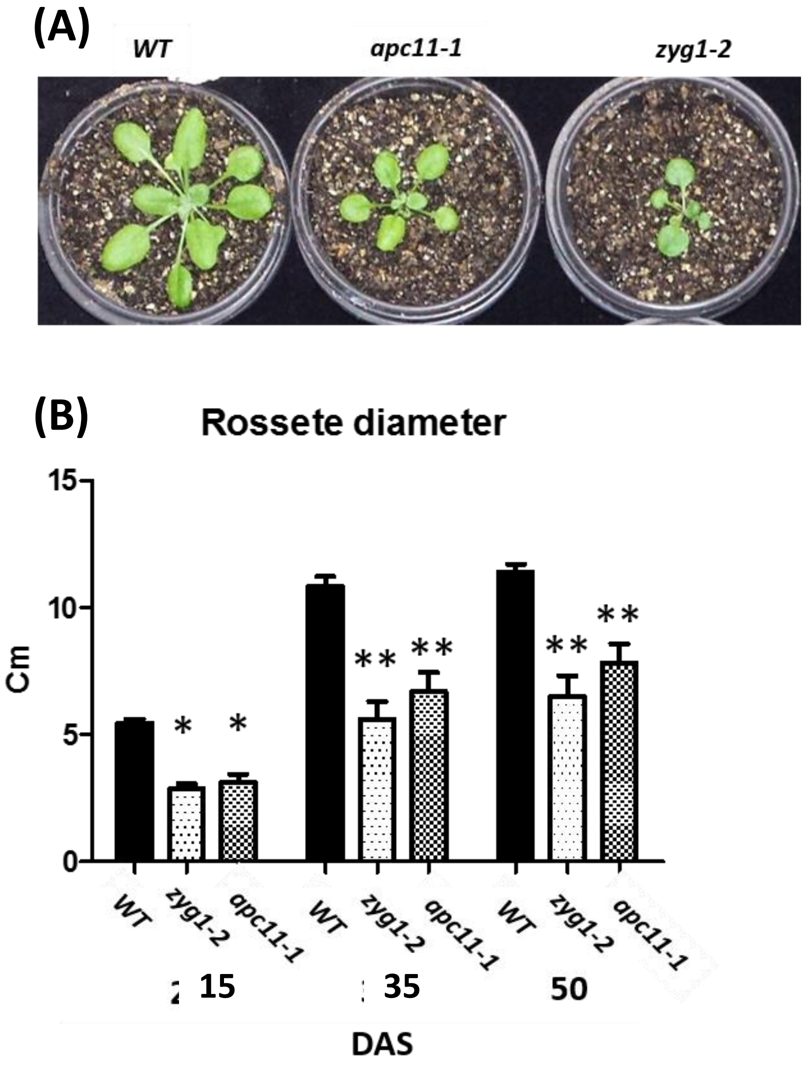

FIGURE S4. ***apc11*** **rosette growth**

Rosette size measurement of the *apc11* SALK mutant lines. The measurements were obtained at 15, 35 and 50 (DAS) and analyzed statistically. Wild type (WT) were used as controls. N = 10. P <0.01 (*), P <0.001 (**) in relation to WT.

(A) Representation of groups in 15 DAS

(B) Graphic representation of *apc11* SALK mutant plants at indicated time points compared to WT.

(A)

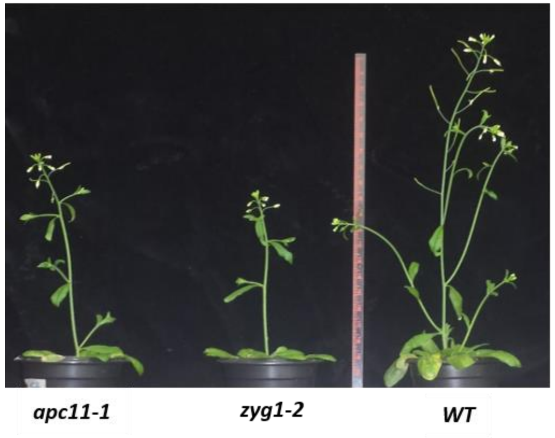

(B)

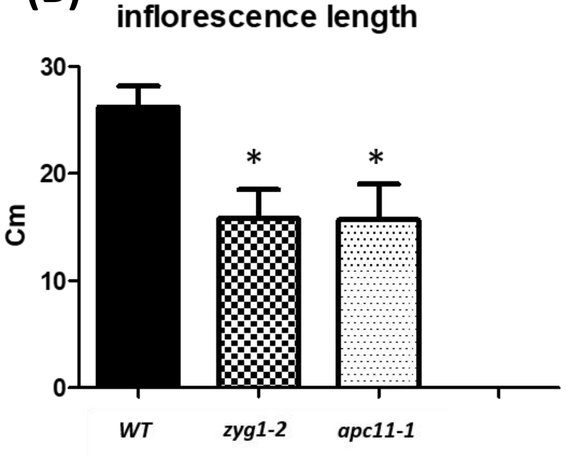

FIGURE S5. *apc11* inflorescence length

Inflorescence length of *apc11 SALK mutants (zyg1-2 and apc11.1*). The measurements were obtained at 50 days after sowing (DAS) and analyzed statistically. Wild type plants (WT) were used as controls. N = 10. P <0.05 (*).

(A) Picture representation of plants at 50 DAS.

(B) Quantitative graphic measurement.

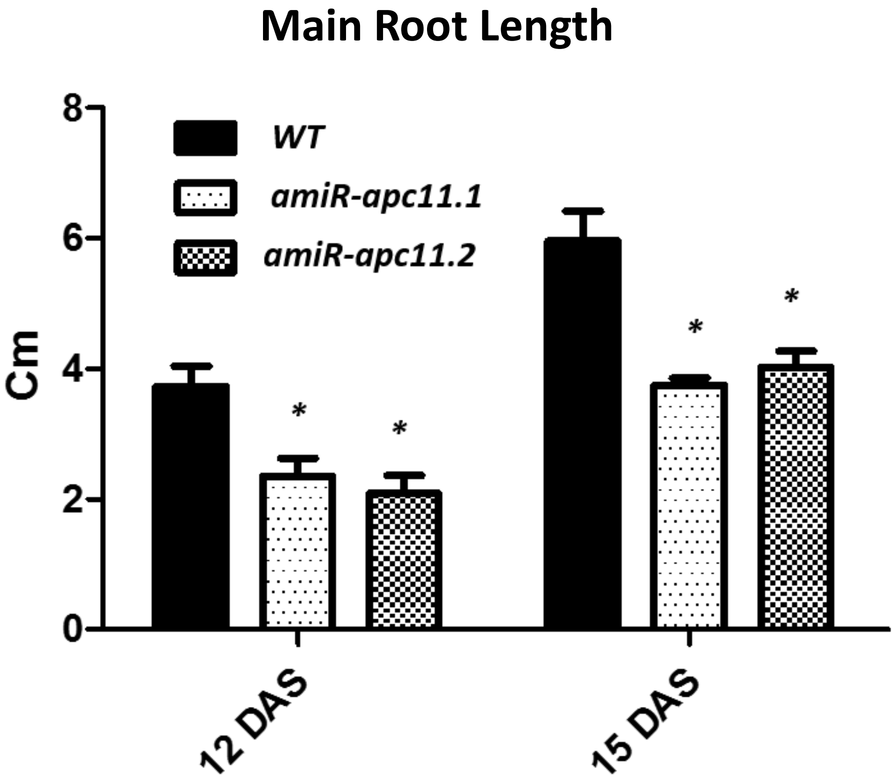

FIGURE S6. *amiR-apc11* develops smaller root size.

Root development in two independent *amiR-apc11.1* and *amiR-apc11.2* lines. Measurement of root length in 12 and 15 DAS. N=8 P< 0.001(*)

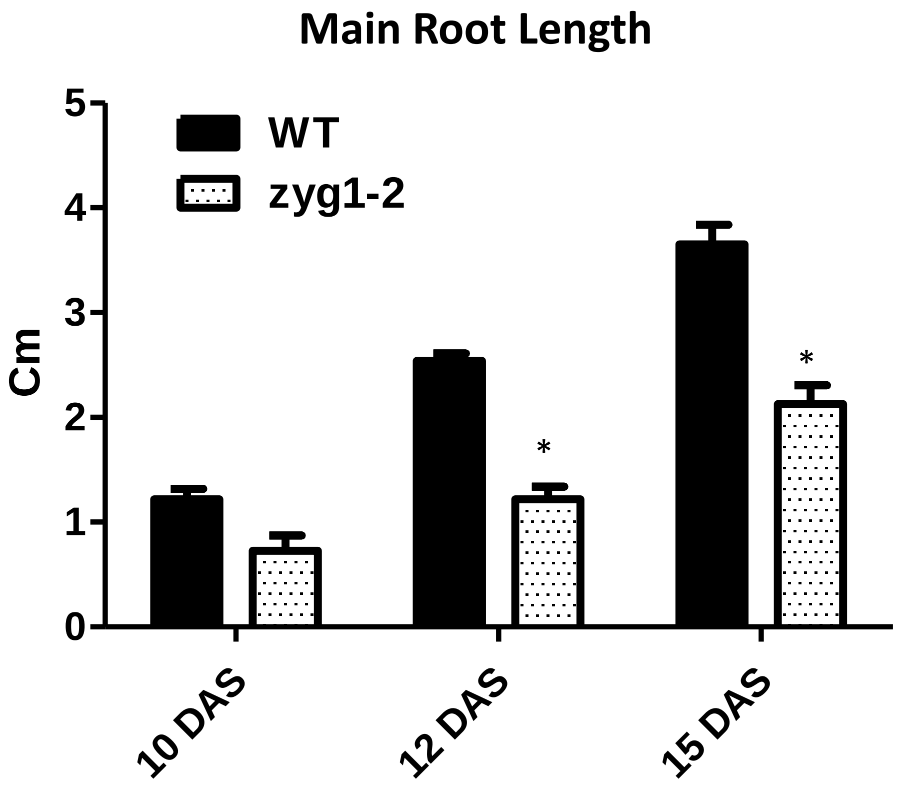

FIGURE S7. *zyg1-2* mutant develops smaller root size.

Root development in *zyg1-2*. Measurement of root length in 10, 12 and 15 DAS. N=8 P< 0.001(*)

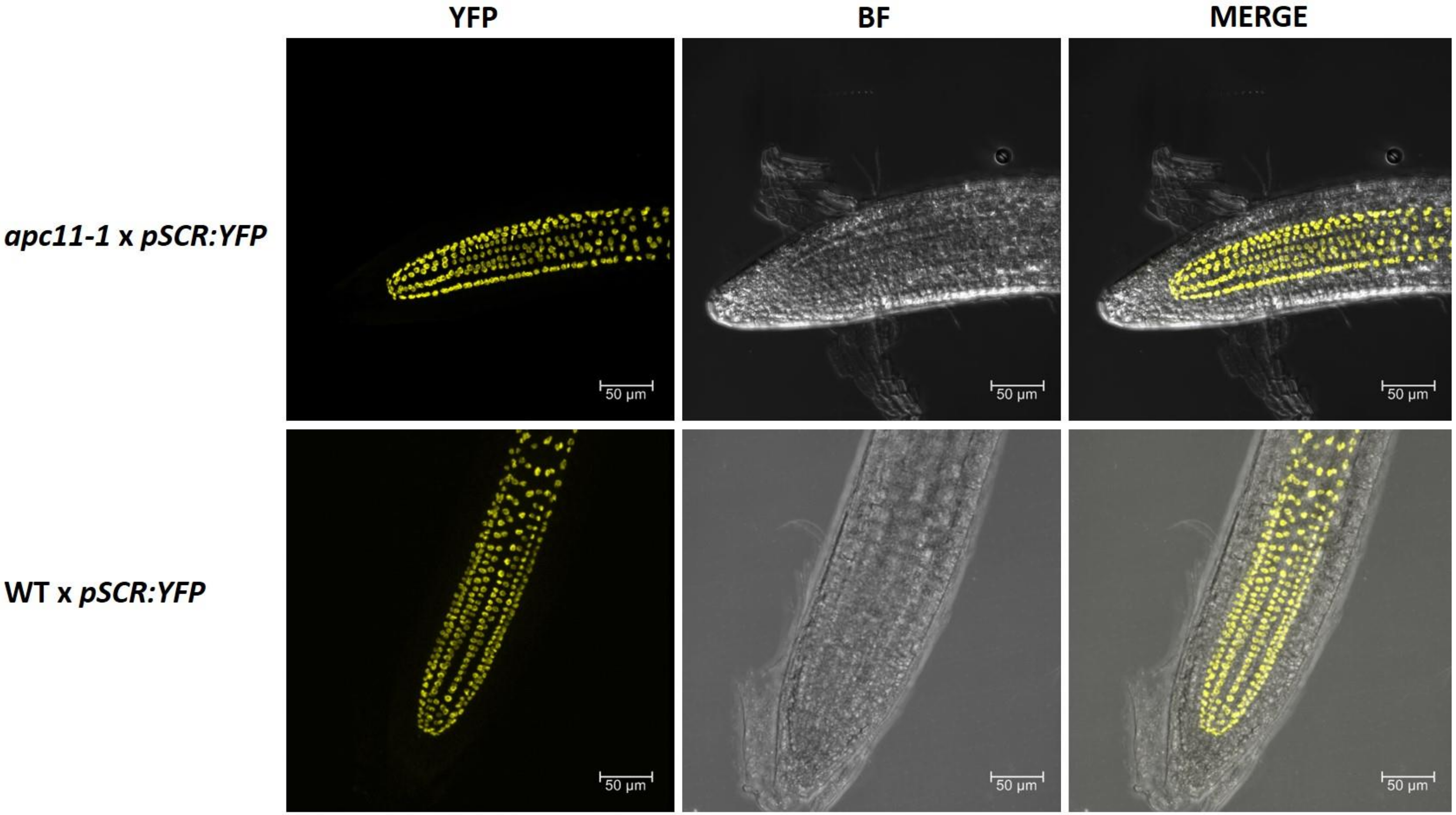

FIGURE S8. *apc11-1* mutant crossed with *pSCR:YFP* marker line. Confocal microscopy of meristematic region of 6 DAS roots of mutant (*apc11-1*) plants crossed with *pSCR:YFP* marker. Right panel, YFP emission. Middle panel corresponds to bright field (BF) images, and right panel the merged picture of YFP and BF.

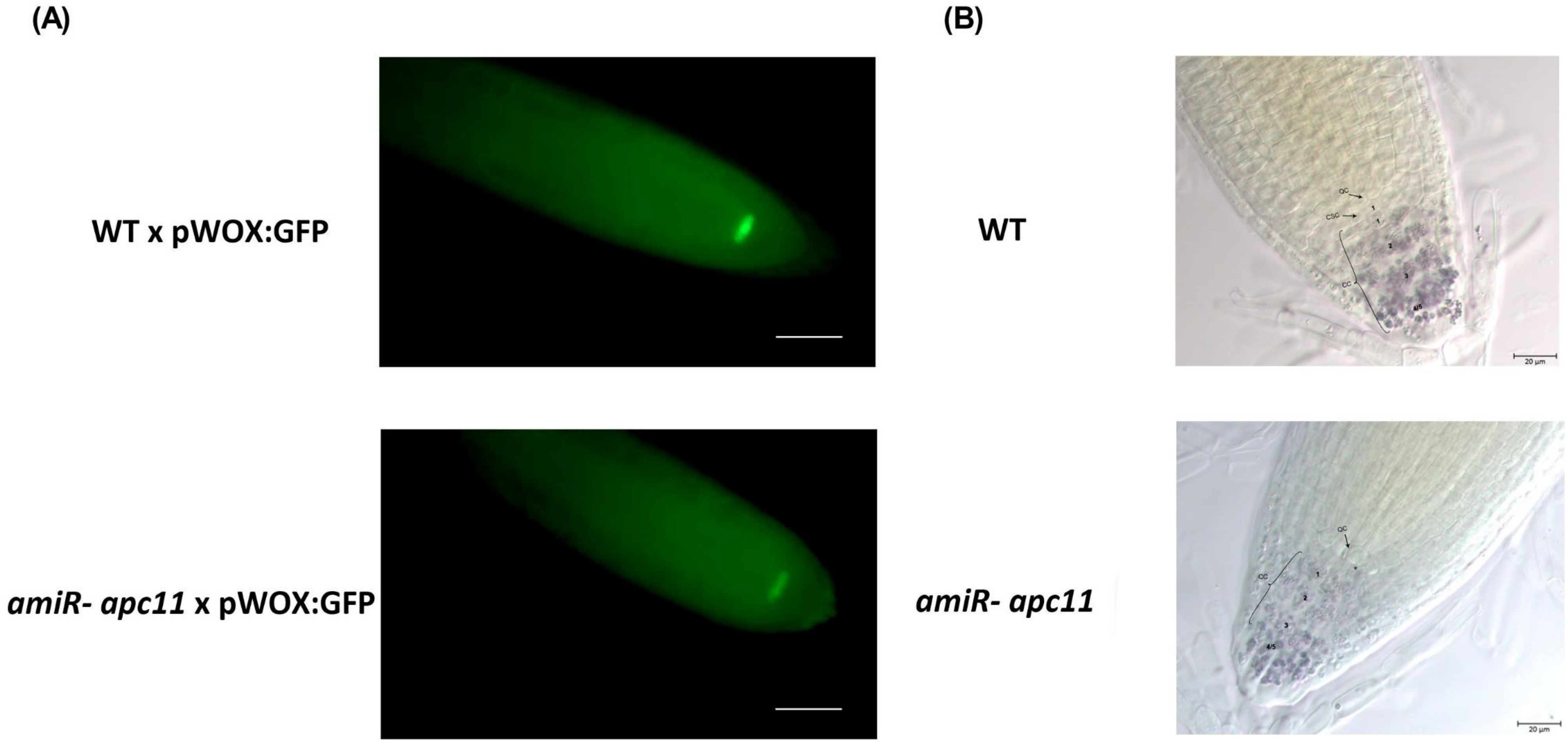

FIGURE S9. *amiR-apc11* crossed with pWOX5:GFP marker

(A) Image representation of root meristematic region at 6 DAS from mutant plants (*amiR-apc11*) crossed with *pWOX5:GFP* marker. Image visualized at Axio Imager fluorescent microscopy. Upper panel - crossing control (WT x *pWOX5:GFP*), and Bottom panel - crossing of *amiR-apc11* x *pWOX5:GFP.* White bar = 50 μM

(B) Representative DIC image of the QC cell in the WT and *amiR-apc11*. Lugol's staining showing the accumulation pattern of starch granules in the columella cells in WT and *amiR-apc11 plants.* *Starch granules indicating the differentiation of Columella Stem Cells (CSC). Black bar = 20 mM

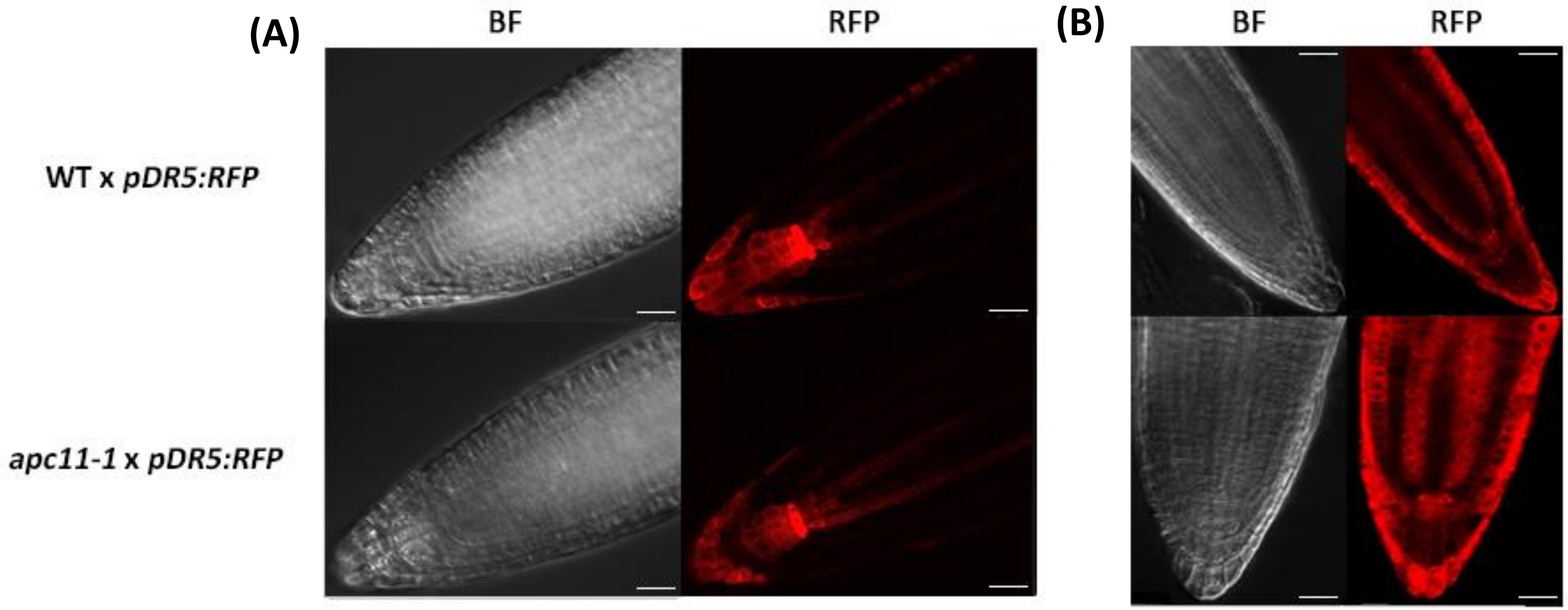

FIGURE S10. ***apc11.1*** **crossed with** ***pDR5:RFP*** **marker**

Confocal microscopy of root meristematic region at 6 DAS from wild type (WT) (upper) and mutant *(apc11-1)* (bottom) plants crossed with *pDR5:RFP*. Right panel RFP emission. Left panel corresponds to bright field (BF) images. Plants show normal phenotype exhibiting quiescent cells expressing DR5:RFP. White bar = 25 μM

(A) Without NAA

(B) With 5μM of NAA

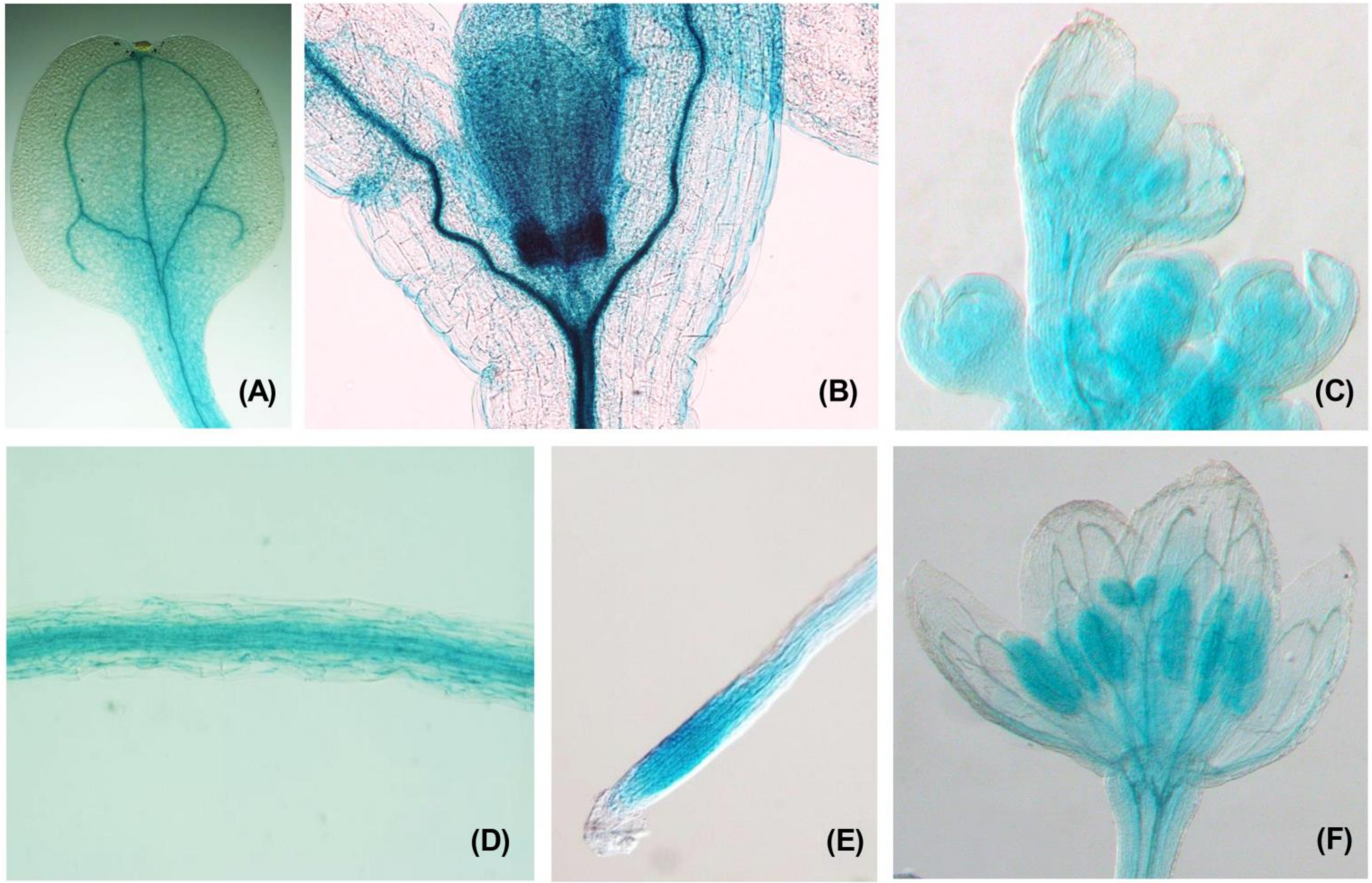

FIGURE S11. ***APC11*** **tissue expression pattern.**

Expression of the p*APC11*–GUS reporter gene at different developmental stages of two independent *APC11* promoter lines.

(A) Cotyledon showing positive marker on vasculature system.
(B) Shoot detail showing staining in meristematic region.

(C) and (F) Flowers bud, and inflorescence

(D) and (E) Main root showing staining on vasculature and meristematic region.

SUPPLEMENTARY TABLE 1 **List of primers used in RT-qPCR experiments and SALK Lines insertion analyses**

Name	Primer Forward Sequence	Primer Reverse Sequence
RT-qPCRs:		
APC11	TTATGGCATGCAGTTGCTTC	GTCTGCGAATTCACCCATTT
CYCD3;1	CCTCTCTGTAATCTCCGATTC	AAGGACACCGAGGAGATTAG
CYCB1;1	GTGCAAAGCTGTTGGCGTAT	GAAGCGTTCGTCCTTGGAGT
CDKB2;1	AGCACACTCAAGAACTGGCA	TGAGATTCGTTTCGCTGGCT
HIS4	CAGCGATTCGTCGTCTTGC	TCCATAGCAGTCACCGTCTTC
Housekeeping genes:		
UBI10	CACACTCCACTTGGTCTTGCGT	TGGTCTTTCCGGTGAGAGTCTTCA
GAPDH	TCCGTGTTCCAACCGTTGAT	GCCTTGGCATCGAAAATGCT
APC 11 Salk Lines:		
T-DNA border primer (LBb1-3)	ATTTTGCCGATTTCGGAAC	
SALK_046847.33.70.x	TGAAGCTTTTTCATTGGGAAAG	AGGGGATGTTTTGGAATGATC
SALK_019654	TGCGTATCCTTTTGTTGCTTC	CTTTGTCCAAGCAATCAAACC
